# Evidence That Frame of Reference Effects Can Reduce Socially Prescribed Perfectionism

**DOI:** 10.3389/fpsyg.2018.02703

**Published:** 2019-01-09

**Authors:** Ayoub Bouguettaya, Tegan Cruwys, Richard Moulding, Ross King, Ana-Maria Bliuc

**Affiliations:** ^1^School of Psychology, Deakin University, Geelong, VIC, Australia; ^2^Research School of Psychology, The Australian National University, Canberra, ACT, Australia; ^3^School of Social Sciences and Psychology, Western Sydney University, Penrith, NSW, Australia

**Keywords:** context, eating behaviour, eating disorders, social norms, perfectionism, social identity

## Abstract

Socially prescribed perfectionism appears to drive disordered eating behaviour in young women, usually via messages from fellow women. Social psychological research suggests that framing effects can be manipulated to reduce the effect of unhealthy messages. This research used contrasting messages about perfectionism to reduce perfectionism among female dieters. We recruited 147 female dieters (*M*_age_ = 25.11) for a between-subjects experimental study. While completing an online questionnaire, participants were exposed to one of three sets of blog posts, which varied in content and source. These three conditions always had one anti-perfectionism message from a woman. This was presented along with either a high perfection message from a man, a high perfectionism message from a woman, or both of these messages. After reading the blog posts, women were asked to fill out a scale assessing their levels of socially prescribed perfectionism. When participants were exposed to an anti-perfectionism message from a woman, paired with a high-perfectionism message from a man, participants showed lower socially prescribed perfectionism than when both high and anti-perfectionism messages came from two women. These findings imply that strategies designed to reduce socially prescribed perfectionism may benefit from including contrasting messages, as this may shift perceived perfectionism norms. Implications for social interventions are discussed.

## Introduction

Disordered eating is a dangerous spectrum of unhealthy eating attitudes and behaviours, including subclinical and clinical symptoms such as binge eating, restrictive dieting, and self-induced purging behaviours (Herpertz-Dahlmann et al., 2008). A number of studies have indicated disordered eating behaviours are widespread, affecting about 20% of young women (McBride et al., 2013). At the subclinical level, disordered eating is associated with poor wellbeing, and can lead to the development of clinical eating disorders (Stice et al., 2011), which have the highest mortality rate of any psychiatric category (Steinhausen, 2002; Arcelus et al., 2011; Smink et al., 2012; Chesney et al., 2014). This death rate can partially be attributed to the difficulty in treating some eating disorders (EDs; Abbate-Daga et al., 2013; Halmi, 2013). Therefore, developing effective *prevention* strategies is critical. However, traditional prevention strategies have also been limited in their effectiveness (Carter et al., 1997; Mann et al., 1997; Smolak et al., 2013). For example, one prevention programme designed to prevent EDs in young women found that those exposed to the intervention had *higher* disordered eating rates compared to controls (Mann et al., 1997). Another school-based programme showed promise by reducing disordered eating immediately after an intervention, but 6 months later, an increase in disordered eating was recorded (Carter et al., 1997). This is possibly because these prevention strategies mostly target specific behaviours, rather than the precursors to disordered eating (Carter et al., 1997). Newer strategies that address the cognitive and social risk factors in disordered eating are more effective at reducing disordered eating behaviour (Sinton and Barr, 2010). Therefore, identifying and targeting these risk factors is crucial in early intervention/prevention strategies.

In particular, research has indicated perfectionism is a strong risk factor for disordered eating development (Fairburn et al., 1999; Bardone-Cone, 2007), and has been shown to both mediate and moderate the relationship between body dissatisfaction and disordered eating (Welch et al., 2009). In this context, perfectionism can be defined as an “overdependence of self-evaluation on the determined pursuit of personally demanding, self-imposed standards” (Shafran et al., 2002, p. 778). The construct of perfectionism in disordered eating is multifaceted and complex (Stairs et al., 2012). One sub-facet of perfectionism, Socially Prescribed Perfectionism (SPP), refers to the perceived need to achieve a standard or goal set by others (Tissot and Crowther, 2008; Stairs et al., 2012) and this has been argued to drive disordered eating behaviours (Tissot and Crowther, 2008; Stoeber et al., 2017). Perfectionism in general appears to predict poorer responses to therapy (Halmi, 2013), but perfectionistic beliefs also appear to be resistant to change through therapy, and predict relapse (Carter et al., 2004).

Part of the reason for this resistance may be because that SPP is not (only) an individual-level risk factor, but is also the internalisation of a social reality. Women in particular are exposed to numerous messages relating to what it means to be a perfect woman (Hesse-Biber et al., 2006). This may partially explain the high relapse rate in EDs (Herzog et al., 1999; Carter et al., 2004; Stice et al., 2009), as when individuals leave therapy, they return to the same environment that initially reinforced the SPP beliefs. This indicates that SPP may be in fact be a *group norm*, in that SPP beliefs encapsulate *what it means to be one of us*. From this perspective, groups of women dictate what it means to be perfect as a norm in their behaviours and attitudes, and members act accordingly to match the expectations of the group. Conceptualising SPP as a group norm held by women means that the social psychological literature offers novel strategies for how health messages can be tailored to reduce perfectionism among at-risk, dieting women. In support of this suggestion, perfectionists tend not to be perfectionistic across all domains, but rather across a select few domains that they deem to be relevant (Stoeber and Stoeber, 2009), indicating that only select social groups should be considered when attempting to understand perfectionism. Furthermore, there is evidence that suggests that SPP levels can be affected by social context (Mitchelson and Burns, 1998), much like how social norms may be affected by context.

Social norms can affect eating beliefs in a variety of ways. Health beliefs and behaviours (including eating behaviours) are influenced by messages from fellow group members (Mackie et al., 1990; Cruwys et al., 2012). Previous studies have suggested that this occurs, in part, due to the effect of such messages on perceptions of *group norms*, or the regularity in beliefs and actions that are perceived to characterise a social group (Hogg and Reid, 2006; Robinson et al., 2013). Interventions that change normative beliefs have been shown to be effective in influencing health behaviours (Wechsler et al., 2003; Cruwys et al., 2015; Miller and Prentice, 2016). In the past 20 years, social norms marketing – or structured attempts to redefine what people consider to be “normal” – has become a dominant approach to health promotion (Wechsler et al., 2003; Miller and Prentice, 2016). However, normative messages that target unhealthy symptoms and cognitions often ignore two key issues: first, in everyday life, such health messages are unavoidably presented in conjunction with *opposing* messages, often from both in-group and out-group sources (Agostinelli and Grube, 2002). Second, the *context* of these messages can affect their impact (Randolph and Viswanath, 2004).

Being aware of the context of health messages is particularly important in contemporary western society, where ED prevention/early intervention strategies are delivered alongside obesity prevention messages (Oude Luttikhuis et al., 2009). These prevention and early intervention strategies have opposing messages; ED prevention/intervention strategies reduce emphasis on weight (Stice and Shaw, 2004), but obesity prevention strategies often encourage daily weighing (Oude Luttikhuis et al., 2009). Furthermore, this may explain why SPP beliefs are resistant to change in therapy as there is a clear disconnect between the therapeutic message and the social backdrop. While in therapy, these perfectionistic beliefs are challenged (Fairburn and Cooper, 2014), but the social context may encourage these beliefs as part of a “cult of thinness” (Hesse-Biber et al., 2006). To ensure that ED prevention and intervention strategies are effective, it may be useful to tailor therapeutic approaches to be aware of, or even harness, the power of social context to change the normative beliefs that precede ED development.

The effectiveness of a message from an in-group member can be enhanced when presented in conjunction with an opposing message from an out-group member (Matheson et al., 2003; Gaffney et al., 2014). This comparative effect has the effect of shifting perceived group norms, such that they become more polarised away from the direction of the out-group members’ message (David and Turner, 1999). In other words, what it means to be *us* can be affected by what it means to be *them*. While research has examined how the comparative context of messages can shift norms in leadership research (Subašić and Reynolds, 2011; Hogg et al., 2012) and feminism research (David and Turner, 1999), few studies investigate this phenomena in the context of health. Recently, applications of social psychological theory to health phenomena have indicated that conceptualising health messages as attempts to modify norms may be useful in the efforts to reduce problematic unhealthy behaviours and beliefs (Hogg and Reid, 2006; Blanton et al., 2008). Thus, social psychological theory may have utility in improving the effectiveness of normative interventions to improve health (Haslam et al., 2009).

Social psychological research has indicated that contrasting messages in an intra-group context can weaken the power of a normative message, if the message given does not fit the expectations of the target of what it means to be a member (Turner et al., 1994). For example, an extreme feminist will be rejected by women in an intra-group context when a moderate feminist is presented as a contrast, because the extreme feminist does not fit the expected group norms (David and Turner, 1999). However, inter-group contrast effects can change perceived norms more effectively than intra-group contrasts, as “what is us” can be affected by “what is them”. In the same study investigating intra-group contrast, it was found that when women were presented with both a radical feminist statement from a woman, and an antifeminist statement from a man, participants perceived the radical group as being more similar to themselves than when the radical feminist statement was presented alone (David and Turner, 1999). In the health context specifically, one study found that when Britons were presented with information about positive Japanese health behaviours, the participants indicated that they felt British people were unhealthy (Tarrant and Butler, 2011). By contrast, when presented information about negative American health behaviours, participants indicated that British people were quite healthy in comparison (Tarrant and Butler, 2011). Furthermore, participants had stronger dieting intentions when they were exposed to the Japanese message than when exposed to the American message (i.e., an aspirational framing). This means that a message from an *in-group* member (i.e., a person from the same group as the target audience) can be enhanced in effectiveness when contrasted with a counter-normative message from an *out-group* member (i.e., a person from a different group than the target audience).

In this way, it may be possible to use comparative context to change unhealthy perceived group norms, such as SPP. Some research has indicated that SPP can be reduced following ED-focused group therapy (Lloyd et al., 2014), and there is evidence that normative change is a mechanism for change in an ED prevention group programme (Cruwys et al., 2015). However, no study has to date has sought to use comparative contexts to reduce unhealthy perfectionism beliefs. We sought to change SPP in particular because previous studies have indicated that it may be resistant to change in more traditional therapeutic messaging (Bastiani et al., 1995), and may predict relapse in EDs (Bastiani et al., 1995; Herzog et al., 1999; Carter et al., 2004).

Our study aimed to investigate how the context of health messages from a variety of group members may alter their effect on SPP norm beliefs, to aid ED prevention/intervention strategies. Because there is evidence that gender is a social group membership that is central to disordered eating (Hesse-Biber et al., 2006), this study approached SPP as a perceived norm among women (as the pressure to be perfect is perceived to come from fellow women; Bouguettaya et al., forthcoming). As the natural contrast to women is men, we sought to use a gender contrast to create a comparative context to shift SPP beliefs among women.

It was hypothesised that presenting a body positive, *low perfectionism* message from an in-group, paired with an out-group *high perfectionism* message (Context A) will result in lower SPP in comparison to Condition B, where that low perfectionism in-group source is contrasted with another in-group source espousing perfectionism (H1). It was also hypothesised that presenting all three messages (Condition C) would also result in higher levels of perfectionism than Condition A, as the comparative context would no longer align with the gender of the presenters, and only the high perfectionism in-group message would be attended to in Condition C (H2). Because previous research has shown that SPP correlates with disordered eating, it was anticipated that dieting intent would be lowest in the Context A (H3), and that reductions in dieting intent in Condition A (relative to other conditions) would be mediated by SPP (H4).

## Materials and Methods

### Participants and Design

Participants were 160 women aged between 18 and 30 years old from the United Kingdom, United States, Canada, and Australia as part of a larger study (which included questions on self-control and strength of identification as a woman). Participants who failed any attention cheques (i.e., a question asking participants were asked to select the option “strongly agree” on a list of possible responses, and two questions asking participants to recall the source of the blog post, and the content of the blog post) or who completed the study too quickly (i.e., under 5 min) were removed from the sample. This resulted in a final sample of 147 women (*M*_age_ = 25.11; *SD* = 3.08). Participants were recruited through Prolific.ac (a UK-based Amazon M-TURK alternative; *n* = 106) and the host university’s online recruitment system (*n* = 41) as part of a larger study. Of the participants, 90 came from the United Kingdom, 49 from Australia, seven from the United States, and one from Canada. No significant differences on any measure (including internal consistency) between nationalities or sample sources (Prolific versus the host university) were detected. Participants were eligible to participate if they reported dieting at some point (yes/no), and never had an eating disorder. These philtres were chosen to exclude people with eating disorders (for ethical reasons).

This study had a between subjects design, with one manipulated variable: context of messages. Our central dependent variable was SPP (Stairs et al., 2012), but we also included a measure of Self-Oriented Perfectionism (Stairs et al., 2012), as well as eating behaviours though the two measures of the EAT-26 (Garner et al., 1982) to assess past/current eating behaviours and the Dieting intentions scale (Cruwys et al., 2013), to assess intent to engage in dietary restriction.

### Procedure

Participants were invited to participate in a study examining “eating tendencies, group-based beliefs, and perfection”. After consenting, information regarding the participant’s age, gender, location, past dieting behaviour, and whether they had ever been diagnosed with an ED was collected. Participants were then presented with hypothetical blog posts, with different combinations depending on the condition. There were three blog posts, including a photo of the ostensive author (drawn from open source face banks, balanced on the basis of attractiveness ratings; see Bainbridge et al., 2013). Participants were asked to read through the posts carefully, as there would be some questions on the content later. The first post (Appendix A; referred to as “Female-Anti-Perfectionism” here) was said to be by a woman stating that women should accept themselves for who they are overall, including how they look and act (counter to perfectionism, with language drawn from Cognitive Behavioural Therapy-Enhanced broad literature; see Fairburn and Cooper, 2014). The second post (referred to as “Male-Perfectionism”) was by a man stating that women needed to be perfect in every way, including how they looked and acted. The third post (referred to as “Female-Perfectionism”) was a similar underlying message but presented in different wordings endorsing perfectionism. Male-Perfectionism and Female-Perfectionism’s posts were matched on every sentence, with simple variations to ensure that they did not read the same. In Condition A, Female-Anti-Perfectionism and Male-Perfectionism were presented. In Condition B, Female-Anti-Perfectionism and Female-Perfectionism were presented. In Condition C, all three statements were presented. The order of the messages was randomised in all conditions, and all messages were designed to make the gender contrast as salient as possible (although each sentence was carefully matched across statements).

Participants were then asked about the detailed content of the messages, which served as an attention cheque. Subsequently, participants were presented with the perfectionism scales, the Dieting Intentions Scale, and the Eating Attitudes Test-26, and other scales from the larger study (in that order). Participants from the host university received course credit for participation, while participants from Prolific.ac received the equivalent of $2.50 for their time. This study was approved by the host university’s ethics panel.

### Materials

#### Measures of Constructs Underlying Perfectionism-Modified (M-CUP)

Socially prescribed perfectionism was measured via a modified version of the Perceived Pressure from Others subscale of the M-CUP (Stairs et al., 2012). This five-item scale originally stated the pressure of “others,” but because our study sought to understand how a specific social group may affect SPP, this scale was changed to specify the pressure as coming from “other women”. This scale’s modified format will be referred to as fSPP to distinguish from the original SPP scale. Self-Oriented Perfectionism was measured via the original High Standards subscale of the M-CUP. This scale assessed the tendency to set high standards for oneself. Both scales request participants to indicate their agreement with the statements presented, on a scale from 1 (strongly disagree) to 7 (strongly agree).

#### Dieting Intentions Scale (DIS)

Future dieting intent was measured via the DIS (Cruwys et al., 2013). This scale measures intention to diet in the next 3 months. This contains seven questions (e.g., “In the next 3 months, I intend to reduce my calorie/KJ intake”) and is assessed on a scale from 1 to 7 (e.g., strongly disagree to strongly agree). This scale was chosen as dieting patterns (dietary restriction) predict disordered eating behaviour and ED development, and can be a form of sub-threshold disordered eating (Thompson and Stice, 2016).

#### Eating Attitudes Test-26 (EAT-26)

Disordered eating was measured through the total score on the EAT-26, a screening tool designed to detect disordered eating behaviours (Garner et al., 1982). The primary use of this scale was to cheque that the groups did not naturally differ on this measure of disordered eating, as it measures past and current behaviours. This scale has 26 statements which describe past and current eating styles. Participants are requested to indicate how frequently they engaged in these behaviours, from a scale from 1 (never) to 5 (always). The response scale was modified from the original 1–6 response scale (where scores below 4 were interpreted as 0), as we wished to detect general frequency of past disordered eating (Mintz and O’Halloran, 2000). The sum of these responses was used in this experiment.

#### Planned Statistical Analysis

The full data set can be found in the Supplementary Materials (as Supplementary Table 1). To test H1 and H2, an ANOVA and a pair of between-subjects *t*-tests (separated based on condition) were performed, with the dependent variable of fSPP. An ANOVA was performed to test H3 (testing Condition A compared to Conditions B and C) on the dependent variable of the DIS. A power analysis using G^∗^Power (Faul et al., 2007) indicated a total sample of 159 people would be needed to detect a medium effect size (*f* = 0.25) with 80% power for an ANOVA with three groups (α = 0.05). A mediational analysis was also performed to test for H4, with the SPSS PROCESS macro using 5000 bootstrap samples (Preacher and Hayes, 2008).

## Results

### Correlations, Descriptive Statistics, and Assumption Checking

Descriptive statistics are presented in Table 1. Statistical analysis was performed via SPPS Version 24.00.00.1. Prior to performing any statistical analyses, relevant assumptions (normality and internal consistency) were checked, with no violations detected. Tests for multivariate outliers were also performed, with none detected (after removing participants who failed attention cheques). Correlations are presented in Table 2. Dieting intent scores in our sample (*M* = 5.38, *SD* = 1.17) were significantly higher than a previous (pre-intervention) sample of dieters (*M* = 4.89, *SD* = 1.19, *N* = 112) using this scale [*t*(257) = 3.32, *p* < 0.01; Cruwys et al., 2015]. The assumption that the groups would not significantly differ on EAT-26 sum scores (or past/current disordered eating behaviour) was supported, as there was no omnibus effect [*F*(2, 144) = 0.704, *p* = 0.496].

**Table 1:** Descriptive statistics (Means, SD, and Cronbach’s Alpha values), *N* = 147.

	M (SD)	Cronbach’s Alpha
Dieting intentions	5.38 (1.17)	0.851
fSPP	4.04 (1.26)	0.924
SOP	5.07 (0.92)	0.869
EAT-26 scores	62.85 (11.61)	0.851
Times dieted in past 6 months	2.96 (2.36)	NA


**Table 2:** Correlations, *N* = 147.

	1	2	3	4
1. Dieting intentions				
2. fSPP	0.208^∗^			
3. SOP	0.197^∗^	0.303^∗∗^		
4. EAT-26 scores	0.425^∗∗^	0.221^∗∗^	0.262^∗∗^	


*^∗^p < 0.05, ^∗∗^p < 0.01.*

### Socially Prescribed Perfectionism (fSPP)

**FIGURE 1:**
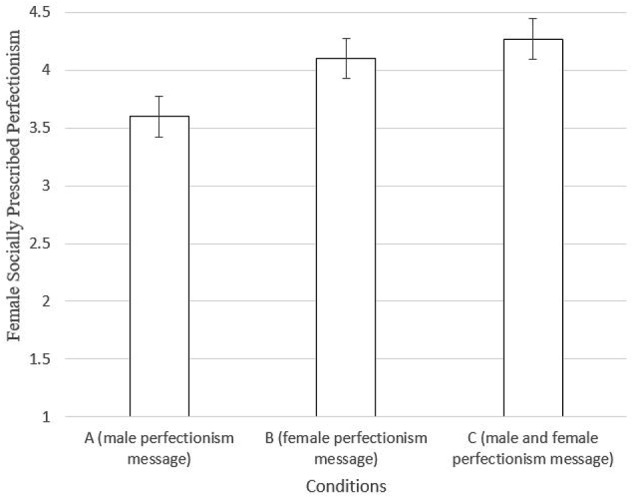
fSPP means between conditions (95% confidence intervals shown). Conditions refers to the contrasting message presented along with the female anti-perfectionism message.

To test H1 and H2, an ANOVA was performed (see Figure 1). A significant omnibus effect was found on fSPP [*F*(2,144) = 3.537, *p* = 0.032]. *Post hoc* tests between groups revealed that Condition A differed from the other two conditions. As per H1, a between-samples *t*-test (Table 3) revealed participants in Condition A showed significantly lower levels of fSPP compared to Condition B (*p* = 0.045; small to medium effect size, Cohen’s *d* = 0.44). H2 was also supported, as the difference between condition A and C was significant (*p* = 0.011, *d* = 0.55), see Table 3. Conditions B and C were not significantly different [*t*(106) = 0.654, *p* = 0.514].

**Table 3:** Between samples *t*-test results comparing Condition A to B and C on fSPP (two-tailed).

Condition	n	Mean (SD)	*T*-value (Comparing to condition A)	95% confidence interval upper, lower
Condition A	39	3.60 (1.10)		
Condition B	49	4.11 (1.21)	-2.03 (*p* = 0.045)	-0.010, -1.001
Condition C	59	4.27 (1.34)	-2.58 (*p* = 0.011)	-0.156, -1.182


To help inform our interpretation of whether fSPP rose or fell, we utilised normative data gathered on the fSPP scale from a questionnaire-based study using the same recruitment source and sample specifications (see Bouguettaya et al., unpublished), and used between-sample *t*-tests to compare to levels of fSPP. This study had an average fSPP score of 4.16 (*SD* = 1.29, *N* = 120). This analysis suggested that fSPP was significantly lower in condition A compared to this de facto control group [*t*(74) = 2.663, *p* = 0.01, 95%CI (0.142, 0.986)], but this de facto control did not significantly differ for condition B [*t*(94) = 0.258, *p* = 0.800, 95%CI(-0.361, 0.469)], nor for condition C [*t*(113) = -0.504, *p* = 0.615, 95%CI(-0.523, 0.311)]. This adds credence to the notion that fSPP was lower among participants in condition A, and not affected by conditions B or C.

### Disordered Eating and Dieting Intentions Between Groups

A second ANOVA was performed to test H3, which stated that dieting intentions would be different between conditions. There was no omnibus effect [*F*(2,144) = 0.20, *p* = 0.82] for dieting intent. Therefore, H3 was not supported.

### SPP Mediation

To test H4’s prediction that fSPP would mediate the link between the manipulations (condition A versus others conditions together) and dieting intent, a mediation analysis was performed. The indirect effect is considered significant when the 95% Confidence interval (CI) does not include zero. While a significant indirect effect was found [β = 0.12, 95% CI (0.02, 0.28)], the direct effect was insignificant (β = -0.01, *SE* = 0.22, *p* = 0.96), as was the total effect (β = 0.11, *SE* = 0.22, *p* = 0.61). Therefore, conditions for mediation were not met (Baron and Kenny, 1986), and H4 was not supported.

## Discussion

The current study investigated how manipulating the context of health messages could change their effectiveness by targeting a specific norm (perfectionism) implicated in disordered eating. There were three main findings. First, health messages from an in-group member appeared to be more persuasive when presented in the context of a dissenting voice from an out-group member (consistent with H1). This in turn, is consistent with previous work which found that the persuasiveness of health messages can be enhanced if the message comes from an in-group members (Mackie et al., 1990; Cruwys et al., 2012), and that comparisons to out-group members can change normative beliefs about health behaviours (Tarrant and Butler, 2011). However, this is the first study to seek to change unhealthy norms regarding perfectionism within groups by contrasting a healthy message against an unhealthy message from an out-group member. This study provides evidence that this social approach may be a promising new avenue for reducing perfectionism.

Second, and consistent with H2, when both a man and woman separately endorsed a perfectionism norm, along with the low perfectionism message from a woman, fSPP scores were greater than a similar condition which omitted the female-perfectionism message. This suggests that dissenting messages from within the in-group may underline the effectiveness of a health message, because the comparative context no longer align with the group membership of the presenters. Instead, the data suggests that participants saw this as an intra-group comparison, with the man effectively ignored in the third condition.

Third, this study sought to reduce dieting intent though reducing fSPP. While it was found that fSPP did weakly correlate with dieting intent (H3), dieting intent was not reduced through reducing SPP norms. This finding does, however, suggest that any social approach to reduce disordered eating through SPP reduction may have to consider more holistic approaches. To do so, it may be critical to understand what perfectionism norms contain in specific social identities for each person and tailor the messages accordingly.

### Implications

These data suggest that because specific forms of SPP can be affected by social context (Mitchelson and Burns, 1998), SPP is not an invariable characteristic of the individual. Rather, SPP can be understood as a *prescriptive, perceived norm* that may relate a social reality (a collective norm) in specific groups of women to emphasise and reinforce a perfectionism norm (Downey and Chang, 2007). This may be the reason why SPP is so difficult to treat in EDs (Bardone-Cone et al., 2007). Traditional individualised approaches have mixed success, and there is some evidence that they can effectively reduce SOP in DEs (Lloyd et al., 2015), but if SPP is a perceived social norm, it is likely to resist redefinition outside of a group context. Consistent with this assertion, here we demonstrated that SPP can be reduced through contextual messages. Therefore, this study provides support for investigating the utility of social psychological approaches for enhancing clinical interventions, as this may provide a path for reducing otherwise resistant and pertinent social beliefs that maintain clinical disorders. Integrating a social intervention method (by adding contrasting messages) in the context of ED therapy may prove a valuable addition to traditional individualised methods for addressing perfectionism.

More broadly, the current study has implications for understanding how effective health messages can be created. Although most health messages do incorporate normative content (Campo et al., 2004), very few incorporate opposing messages. This is despite evidence suggesting that the presentation of opposing groups can change health behaviours (Tarrant and Butler, 2011). This study suggests that crafting health messages to change perceived norms may need to be aware of context in order to increase the effectiveness of existing therapeutic methods.

### Limitations

There are several limitations in this study. First, this study did not include conditions where the messages were presented by themselves, nor was there a context where the male and the female presented their similar messages together. This was largely due to ethical considerations, as we did not wish to increase perfectionism in females with dieting tendencies. Therefore, uncertainty exists as to the effectiveness of each individual message. For the same reason, these data cannot distinguish whether the mere presence of the male perfectionism message reduced perfectionism in the predicted direction, or whether participants ignored the male and only attended to the female anti-perfectionism message in Condition A, nor can it directly ascertain that there was a decrease in perfectionism in Condition A (only indirectly, as compared to previous studies). There is evidence to suggest that people process messages from out-group members differently (Vonk and van Knippenberg, 1995). Consistent with this, near identical messages from a man versus from a woman were attended to differently in this study; in condition A, the perfectionism message from the man was ignored, while in condition B, the perfectionism message from the woman was influential. This indicates that it is the source, rather than its content, that was the primary driver of social influence in this study. This study was unable to reduce disordered eating through reducing fSPP as well. This may be due to the smaller than expected relationship of fSPP and disordered eating in this casual dieting sample, which may be due to the greater disconnection than anticipated between fSPP and dieting. More specifically, the nature of perfection definitions within this identity may mean that perceived norms for perfection as a woman may not be related to perceived dieting norms (e.g., the definition may instead relate to fitness, rather than dietary restriction). Instead, future research may wish explore how this method could be used with a sample with high fSPP and high disordered eating. However, this study did show that reducing fSPP is possible, and as SPP in general can be a risk factor to future disordered eating, it is still possible our study had an effect on future actual disordered eating behaviours (not just on dieting intent).

## Conclusion

The present study illustrates that the effectiveness of health messages from an in-group source may need to be context-aware, as contrasting messages may increase decrease the power of these messages. However, at the present stage most, if not all, social health messages only present messages from one source at a time. Perceived group norms impact health in a number of ways, and while some research has shown that implicit comparisons can change perceived group norms (Tarrant and Butler, 2011), this study is the first to present how direct comparisons can change group health norms in a desired direction. This study argues that the inclusion of out-group members in health messages may be a plausible, gainful method to increase the effectiveness of existing messages, in line with inoculation theory research on resistant health attitudes (Compton et al., 2016). Integrating this social identity approach with inoculation theory may provide result in improved interventions as well, as social identity approaches may be able to specify contextual effects that lead to effective inoculation (Tarrant and Butler, 2011). This study also provides a potential avenue for changing perfectionism, which is a belief that is resistant to change and implicated in a serious condition (EDs; Carter et al., 2004). Specifically, our study argues that the context of an anti-perfectionism message may matter in therapy, as an anti-perfectionism message from a woman was only influential in a condition without a pro-perfectionism message from another woman. In sum, this study suggests that harnessing the power of group dynamics may be a practical method for using the power of groups to benefit health.

## Ethics Statement

This study was carried out in accordance with the recommendations of Deakin University Human Research Ethics Committee at Deakin University with written informed consent from all subjects. All subjects gave written informed consent in accordance with the Declaration of Helsinki. The protocol was approved by the Deakin University Human Research Ethics Committee.

## Author Contributions

AB conceived the study, recruited participants, organised the data, performed the statistical analysis, and wrote the first draught of the manuscript. TC and A-MB were helped to guide the social psychological concepts used, with the TC providing alternative theoretical explanations. A-MB aided with recruitment. RM and RK were provided conceptual help from a clinical perspective, helping to integrate key clinical concepts into the draught based on experience. RM provided statistical expertise. All authors revised, read, and approved the present submitted version.

## Conflict of Interest Statement

The authors declare that the research was conducted in the absence of any commercial or financial relationships that could be construed as a potential conflict of interest.
