# Supplementary material for: Evidence That Frame of Reference Effects Can Reduce Socially Prescribed Perfectionism
Source: Front Psychol. 2019 Jan 9;9:2703. doi: 10.3389/fpsyg.2018.02703 (PMC6333663; doi:10.3389/fpsyg.2018.02703)
Supplement: Supplementary file 1 [file Table_1.docx]

Appendix A: Anti-Perfectionism message from a woman

Hi friends ☺

For years, I thought about what it meant to be the perfect woman. I kept having “shoulda, coulda, woulda” moments: I kept thinking about what I should be, what I could be, and what I would be if I only could work what I should, and I realised I fell into a trap! Today, I’m going to talk to you about how I got out of the trap of wanting to be perfect in every way, and started loving me for me!

One of the worst things about wanting perfectionism is that you’ll never get out of the trap. There’s always something higher, and taller- and you’ll very quickly realise it. My struggle for perfection- and I believe, most women see this- was all about how I looked and how much I weighed. Then I realised the “should” was taking over my life and hurting the people around me- “I should weigh X number of pounds. If I don’t, no one will find me attractive or lovable. I should always look put together. If I don’t, people will think I don’t care. They’ll reject me.” So every day I’d have a diary of how much I ate, how much I ran, and what I ate, and worried about where it would go. My hips, my ankles, and wrists…. I worried what my boss would say or even strangers! To me, if I wanted to be perfect, I needed to control these things, be strong, so I could show my bikini bod without worry.

I ended up being steamrolled into a situation it had nothing to do with reality! Perfect then, was all or nothing; even a gram over my target meant I was not perfect. And even if I did hit my goal, I would look at myself and be upset- there was always a hair out of place.

So I made a change. I thought, let’s see what happens. I replaced my diary with my “should” statements, and went out and tested them. My first should was, “I should weigh 2 kilos less to be perfect, or people won’t love me.” My “for” evidence was: “People will think badly of me if they see me now.” My “against” evidence might be: “Being this weight has no effect on how people see me” and “This rule makes me feel bad about myself all week if I didn’t lose those kilos.”

Then I revised it to a more balanced one. Instead of a demand, I looked at it as a preference. For instance, “I prefer to be 2 kilos lighter, but sometimes that might make it harder to be perfect at work.”

If you notice that your “should” statements are absolute, then you might want to reconsider. After all, perfection is an attitude, not a result. If you want to be a perfect woman, you gotta remember that there’s more to life than self-control and strength in body shape, but more in who you are. Be the best friend or mother or worker instead, and recognise you can be more effective if you realise them as preferences, not demands. You’ll come out healthier and happier for it, like me!

If you like these tips, let me know- and together, let’s be the best ladies we can be- be letting us be US!
